# Identification of rice landraces with promising yield and the associated genomic regions under low nitrogen

**DOI:** 10.1038/s41598-018-27484-0

**Published:** 2018-06-15

**Authors:** I. Subhakara Rao, C. N. Neeraja, B. Srikanth, D. Subrahmanyam, K. N. Swamy, K. Rajesh, P. Vijayalakshmi, T. Vishnu Kiran, N. Sailaja, P. Revathi, P. Raghuveer Rao, L. V. Subba Rao, K. Surekha, V. Ravindra Babu, S. R. Voleti

**Affiliations:** ICAR-Indian Institute of Rice Research, Rajendranagar, Hyderabad 500030 India

## Abstract

With the priority of the low input sustainable rice cultivation for environment friendly agriculture, NUE of rice becomes the need of the hour. A set of 472 rice genotypes comprising landraces and breeding lines were evaluated for two seasons under field conditions with low and recommended nitrogen and >100 landraces were identified with relative higher yield under low nitrogen. Donors were identified for higher N uptake, N translocation into grains and grain yield under low N. Grains on secondary branches, N content in grain and yield appears to be the selection criterion under low N. Through association mapping, using minimum marker set of 50 rice SSR markers, 12 genomic regions were identified for yield and yield associated traits under low nitrogen. Four associated genomic regions on chromosomes 5, 7 and 10 were fine mapped and QTL for yield under low N were identified from the marker delimited regions. Three candidate genes viz., 2-oxoglutarate /malate translocator (Os05g0208000), alanine aminotransferase (Os07g0617800) and pyridoxal phosphate-dependent transferase (Os10g0189600) from QTL regions showed enhanced expression in the genotypes with promising yield under low N. Marker assisted selection using SSR markers associated with three candidate genes identified two stable breeding lines confirmed through multi-location evaluation.

## Introduction

Rice is cultivated in an area of 163.1 million ha with ~15% of global nitrogen (N) fertilizer inputs resulting in the production of 748 million tonnes (FAO, 2016) (www.fertilizer.org). However, the input use efficiency of N is low with ~30–50%, increasing the cost of cultivation and environmental pollution^1^. Thus, nitrogen use efficiency (NUE) in rice becomes important through the nitrogen application management strategies as well as through the development of varieties with high NUE. Genetic variation for NUE in rice germplasm has been earlier reported^2–4^. Several rice genotypes were studied for yield and NUE through field evaluation and efforts were made to characterize the correlations of agro-morphological traits and parameters with yield and NUE^2–8^. NUE including N uptake, translocation, assimilation, and remobilization is inherently complex and is governed by multiple genetic and environmental factors^9^ and several quantitative trait loci (QTL) have been reported using many bi-parental populations for N metabolism and NUE in rice^10^.

Rice landraces adapted to the local environments and selected by the farmers for their better yield under low or zero inputs form an interesting genetic material for identification of donors and genomic regions for better NUE. Landraces, given their past evolutionary history and adaptation to stress environments, often out-yield modern cultivars under low-input production systems^11^.

Association mapping based on linkage disequilibrium (LD) is a high-resolution method for simultaneous detection of multiple loci and their alleles associated with complex traits. LD is the non-random association of alleles at different loci, caused by the correlation between the allele polymorphisms (of markers) due to their shared history of mutation and recombination. Selection, mutation, migration, linkage and admixture increase LD^12^. The indirect selection by farmers over time would have increased the LD of rice landraces, which can be exploited for association mapping. Using simple sequence repeat (SSR) markers, several agronomically important traits including NUE have been identified through association mapping in rice^13–15^. Recently, Genome-Wide Association Study (GWAS) has become a powerful tool in rice to identify genomic regions associated with traits of interest using Single Nucleotide Polymorphism (SNPs)^16–18^.

Since, NUE is finally calculated on yield basis, the information about the variability of yield and yield related components in the rice germplasm under low N situation is important for developing rice varieties with NUE. Thus, using rice landraces adapted to low inputs and strategy of association mapping, an attempt was made in the present study to identify promising donors for yield and the associated traits/parameters and genomic regions for yield under low N through i) evaluation of 472 genotypes comprising landraces and breeding lines under field conditions in wet (WS) and dry seasons (DS) with low and recommended N; ii) characterization of correlations of nine agro-morphological traits and parameters under low N iii) assessment of panicle component traits contributing towards yield under low N and v) to identify genomic regions associated with yield under low N using a strategy of minimum set of 50 polymorphic rice SSRs across 12 chromosomes through association mapping and fine mapping of a few associated genomic regions as a proof of concept.

## Results

### Identification of promising donors for yield under low N

Wide variation with a general trend of reduction for the nine agro-morphological traits and parameters was observed in 472 genotypes under low N compared to recommended N across the seasons under field evaluation (Table 1) (Supplementary Fig. S1). Significant differences among genotypes were found by ANOVA for all the parameters but for the panicles m^−2^. N treatment was found to be significant only for grain yield. In the present study, NUE was analysed using three indices *viz*., physiological NUE (PNUE kg kg^−1^) as total dry weight/total N uptake to study conversion efficiency, internal efficiency (IE kg kg^−1^) as grain yield/total N uptake and NHI (%) as N in grain/N in plant to study partitioning efficiency. For three NUE indices, despite the wide range of values obtained, N treatment was not significant, whereas significant genotypic variation was observed (Table 1). The interaction between treatment and genotypes was significant for plant height, total N uptake and NUE indices. Significant differences were also detected for all the interactions between genotypes and seasons and also among genotypes, treatments and seasons. The analysis of variability for seven additional traits *viz*., SPAD during vegetative stage, number of tillers per hill, number of productive tillers per hill, straw weight kg ha^−1^, total dry matter kg ha^−1^, N in grain kg ha^−1^ and N in straw kg ha^−1^ under low N compared to recommended N were presented in Supplementary Table S1.

**Table 1:** Summary of ANOVA of agro-morphological traits, N content and NUE indicators of 472 genotypes under low and recommended N in field during wet and dry seasons

Traits/Parameters	Range				Mean				T	S × T	G	S × G	T × G	S × T × G
	Wet		Dry		Wet		Dry							
	Low N	Rec N	Low N	Rec N	Low N	Rec N	Low N	Rec N						
SPAD RS	10.10–38.95	10.08–45.91	14.80–44.47	13.89–49.59	27.44	30.72	30.2	32.2	ns	**	**	**	ns	**
PH	44.67–150.00	66.33–184.00	36.66–122.67	72.00–154.67	87.1	125.87	75.07	91.4	ns	**	**	**	**	**
PN	128–379	156–431	125–368	174–428	281	344	275	329	ns	ns	ns	**	ns	**
GY	905.28–3424.31	1038.33–5395.53	380.12–3376.67	1240.00–4974.31	2502.09	3700.8	1967.14	2999.5	*	ns	**	**	ns	**
HI	0.19–0.55	0.21–0.58	0.16–0.57	0.27–0.59	0.48	0.49	0.44	0.5	ns	*	**	**	ns	**
TNUP	15.26–60.95	18.15–75.63	15.51–63.06	23.45–76.96	38.59	58.33	31.29	47.27	ns	ns	**	**	**	**
PNUE	94.47–185.54	94.42–189.88	96.59–192.28	100.42–177.56	136.61	131.31	143.31	121.33	ns	*	**	**	**	**
IE	29.59–96.56	35.83–98.68	25.37–97.34	39.46–99.60	63.99	64.97	63.16	64.2	ns	ns	**	**	**	**
NHI%	36.47–83.47	37.74–83.47	23.10–85.73	47.63–82.03	71.45	70.44	67.49	71.99	ns	*	**	**	**	**

SPAD RS: soil-plant analyses development during reproductive stage; PH: Plant height (cm); PN: panicles m^−2^; GY: Grain yield kg ha^−1^; HI: Harvest index; TNUP: Total N uptake kg ha^−1^: PNUE: Physiological N use efficiency kg kg^−1^; IE: Internal efficiency kg kg^−1^; NHI: Nitrogen harvest index; T: Treatments (low and recommended N); S: Seasons (wet and dry); G: Genotypes.*p < 0.05, **p < 0.01

**Figure 1:**
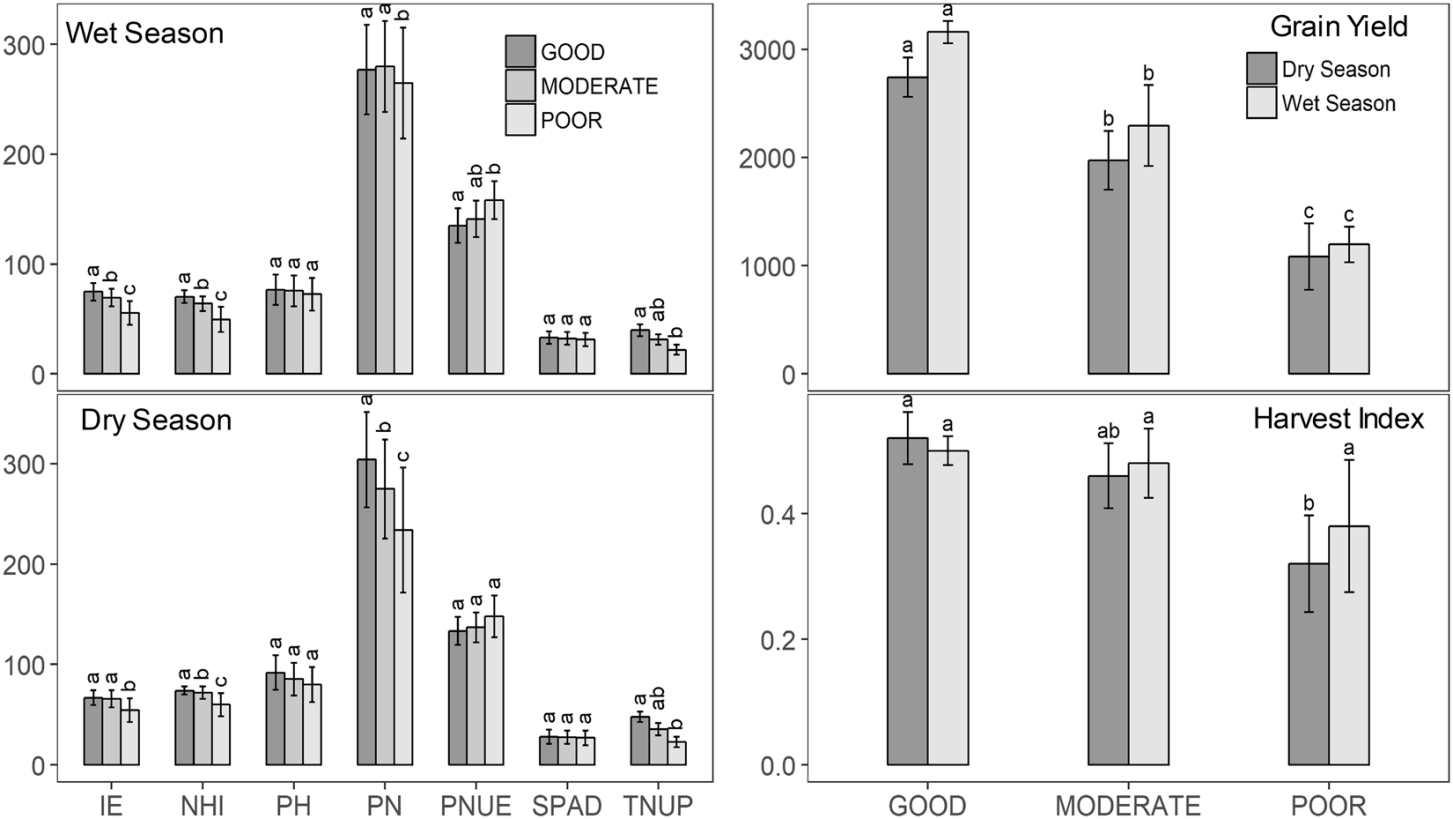
(**a**) Representation of variation of IE kg kg^−1^, NHI%, PH (cm), PN, PNUE kg kg^−1^, SPAD-RS, TNUP kg ha^−1^ in high yielders (>2500 kg ha^−1^), moderate yielders (>1500 kg ha^−1^) and low yielders (<1500 kg ha^−1^) in dry season (**b**). Representation of variation in wet season (**c**). Representation of variation of grain yield kg ha^−1^ in wet and dry seasons (**d**). Representation of variation of HI kg ha^−1^ in wet and dry seasons. Each bar represents the mean of 3 independent replications ± STDEV (means followed by the same letter/s are not significantly different (P > 0.05).

Under low N, minimum mean reduction of plant height by 17.8%; panicles m^−2^ by 16.1%; grain yield by 18.3% and total N content by 33.8% was observed across the seasons. The trend of reduction was also seen for the additional traits like tillers, productive tillers, straw weight, total dry matter and N in grain and straw (Supplementary Table S1). However, some landraces have shown buffering capacity under low N with high number of panicles, total N uptake, total dry matter and yield indicating their efficient uptake and utilization of available N into yield. Analysis of the variation for the nine traits/parameters across the genotypes grouped as high yielders (>3000 kg ha^−1^ - WS) (>2500 kg ha^−1^ - DS), moderate yielders (>1500 kg ha^−1^) and low yielders (<1500 kg ha^−1^) clearly showed significant differences for the total N uptake by the high yielders in comparison to the other two groups. The internal efficiency (kg kg^−1^) and NHI% of the low yielders found to be significantly different from high and moderate yielders (Fig. 1). In both seasons, N in grain found to be differentiating the high, moderate and low yielders (Supplementary Fig. S2).

Thus, 103 donors with promising grain yield >3000 kg ha^−1^ - WS and 122 donors >2500 kg ha^−1^ - DS were identified under low N and 26 genotypes were found to be common across seasons as listed in Supplementary Table S2. As our focus is yield along with NUE, 30 genotypes (WS) and 24 genotypes (DS) with high IE (>68 kg kg^−1^) were further selected among the genotypes with higher yield under low N.

A sub-set of 206 genotypes from the set of 472 genotypes was investigated for the distribution of spikelets and grains across the panicle. Each panicle was divided into upper and lower portion and the spikelet number and grain number was counted on primary and secondary branches. ANOVA showed significant differences for all the traits, but for grain filling % across the panicle (Table 2). For total number of spikelets and grains, significant reduction was observed by 21.9% and 20.4% under low N. Similar reduction was observed in the lower portion of the panicle for number of spikelets by 24.2% and number of grains by 23.1% under low N, indicating adverse affect of limited N supply on lower portion of panicle. The spikelets and grains on secondary branches were observed to be reduced by 25.6% and 24.6% in comparison to the primary branches. The magnitude of reduction for spikelets and grains across the panicle was shown in Table 3. The highest reduction (28.8%) was found for spikelets on the secondary branches and followed by grains on secondary branches (27.6%) of lower portion under low N. In the present study, genotypes were identified with maximum number of spikelets ~135 and grains ~100 on secondary branches in the lower portion of the panicle, which can be deployed donors for grain number under low N. Similarly, genotypes have also been identified with high number of spikelets and grains on primary branches of lower portion and primary and secondary branches of upper portion of the panicle (Table 2).

**Table 2:** Summary of ANOVA for spikelets, grains and grain filling (GF) % across the panicles of 206 genotypes grown under low and recommended N in field.

Trait/Parameter	Range		Mean		F value			LSD		
	Low N	Rec N	Low N	Rec N	T	G	T × G	T	G	T × G
Panicle length	16.7–29.1	17.9–34.2	22.4	25.3	404**	342**	127.5**	0.142	0.443	0.626
Total spikelets	48.5–262.77	66.6–324.3	122.8	157.6	572**	1971**	909**	14.4	2.84	3.99
Total grains	40.9–240.82	58.02–260.5	112.6	141.5	122**	87**	41.2**	25.9	12.1	17.1
Total GF%	79.1–97.6	72.2–97.7	91.5	89.9	NS	2.88**	1.97**	NS	7.08	10.28
Primary branches										
Spikelets	29.5–100.7	30.6–114.8	52.6	63.0	102.1**	309.7**	175.9**	10.25	2.29	3.24
Grains	26.6–82.7	25.1–94.0	47.7	56.2	237.2**	167.2**	80.8**	5.46	2.91	3.98
GF%	69.5–99.1	62.1–99.7	90.9	89.6	NS	10.3**	8.77**	NS	5.34	7.55
Secondary branches										
Spikelets	17.3–185.14	27.9–214.2	70.3	94.5	881**	3475**	1641**	8.11	1.63	2.31
Grains	14.0–106.87	23.4–202.4	64.3	85.3	95.8*	60.9**	29.5**	8.93	11.31	16.00
GF%	81.3–97.5	68.5–97.5	91.8	90.1	NS	1.23*	NS	NS	10.1	NS
Upper portion										
Spikelets	18.7–114.0	25.1–139.6	49.5	60.8	157**	652**	358**	3.44	2.21	3.11
Grains	16.4–105.4	24.2–126.9	45.5	54.2	282.7**	126.9**	68.02	5.15	4.63	6.54
GF%	80.4–97.2	66.9–98.3	91.7	89.2	NS	2.72**	2.34**	NS	7.31	10.35
Lower portion										
Spikelets	28.0–170.8	37.6–197.1	73.4	96.8	450**	1186**	1018**	10.95	1.84	2.60
Grains	22.8–125.4	34.1–169.6	67.1	87.3	67.3*	55.4**	31.5**	10.6	9.6	13.5
GF%	73.6–98.1	70.1–98.6	91.3	90.4	NS	2.72**	1.8**	NS	8.72	12.34
Upper portion – primary branches										
Spikelets	8.4–49.6	12.2–55.6	20.8	24.6	36.6*	195.6**	139.8**	2.69	1.40	2.00
Grains	6.9–42.4	11.7–44.4	18.7	21.4	32.8*	60.6**	30.5**	2.03	2.33	3.29
GF%	69.4–99.4	62.9–99.2	89.8	87.7	70.1*	4.2**	3.8**	1.09	9.45	13.36
Upper portion – secondary branches										
Spikelets	6.5–80.9	12.4–92.7	28.7	36.2	81.7*	669.4**	341**	3.57	1.61	2.38
Grains	5.0–77.4	9.6–97.2	26.8	32.8	75.2*	111.2**	55.6**	3.0	3.76	5.33
GF%	78.7–98.1	48.7–97.8	92.9	89.9	8.01	1.55**	1.15	NS	9.29	NS
Lower portion – primary branches										
Spikelets	15.5–54.0	16.2–73.4	31.8	38.5	37.1*	204.2**	117.9**	4.69	1.81	2.57
Grains	13.2–54.0	12.5–61.1	29.1	34.8	411.0**	255.1**	128.1**	2.83	1.48	2.09
GF%	62.8–100.0	60.4–100.0	91.6	91.3	0.401	9.38**	8.45**	NS	6.29	8.88
Lower portion – secondary branches										
Spikelets	8.9–134.2	15.2–151.5	41.5	58.3	260.0**	44698**	25672**	0.325	0.29	0.415
Grains	7.3–100.4	13.2–116.9	38.0	52.5	34.6*	37.2**	22.1**	10.5	9.12	12.97
GF%	78.6–98.8	70.9–98.4	90.9	89.9	NS	1.22*	NS	NS	9.68	NS

T: Treatments (low and recommended N); G: Genotypes *p < 0.05, **p < 0.01.

**Table 3:** The magnitude of reduction of panicle components under low N in comparison to recommended N.

Panicle traits/Parameters	Reduction (%)
Spikelets on secondary branches of lower portion	28.8
Grains on secondary branches of lower portion	27.6
Spikelets on secondary branches	25.6
Grains on secondary branches	24.6
Spikelets of lower portion	24.2
Grains of lower portion	23.1
Total spikelets	22.1
Spikelets on secondary branches of upper portion	20.7
Total grains	20.4
Spikelets of upper portion	18.5
Grains on secondary branches of upper portion	18.2
Spikelets on primary branches of lower portion	17.4
Spikelets on primary branches	16.5
Grains on primary branches of lower portion	16.4
Grains of upper portion	16.1
Spikelets on primary branches of lower portion	15.4
Grains on primary branches	15.1
Grains on primary branches of upper portion	12.6
Panicle length	11.5

### Positive correlation of SPAD and panicles m^−2^ with high yield under low N

**Figure 2:**
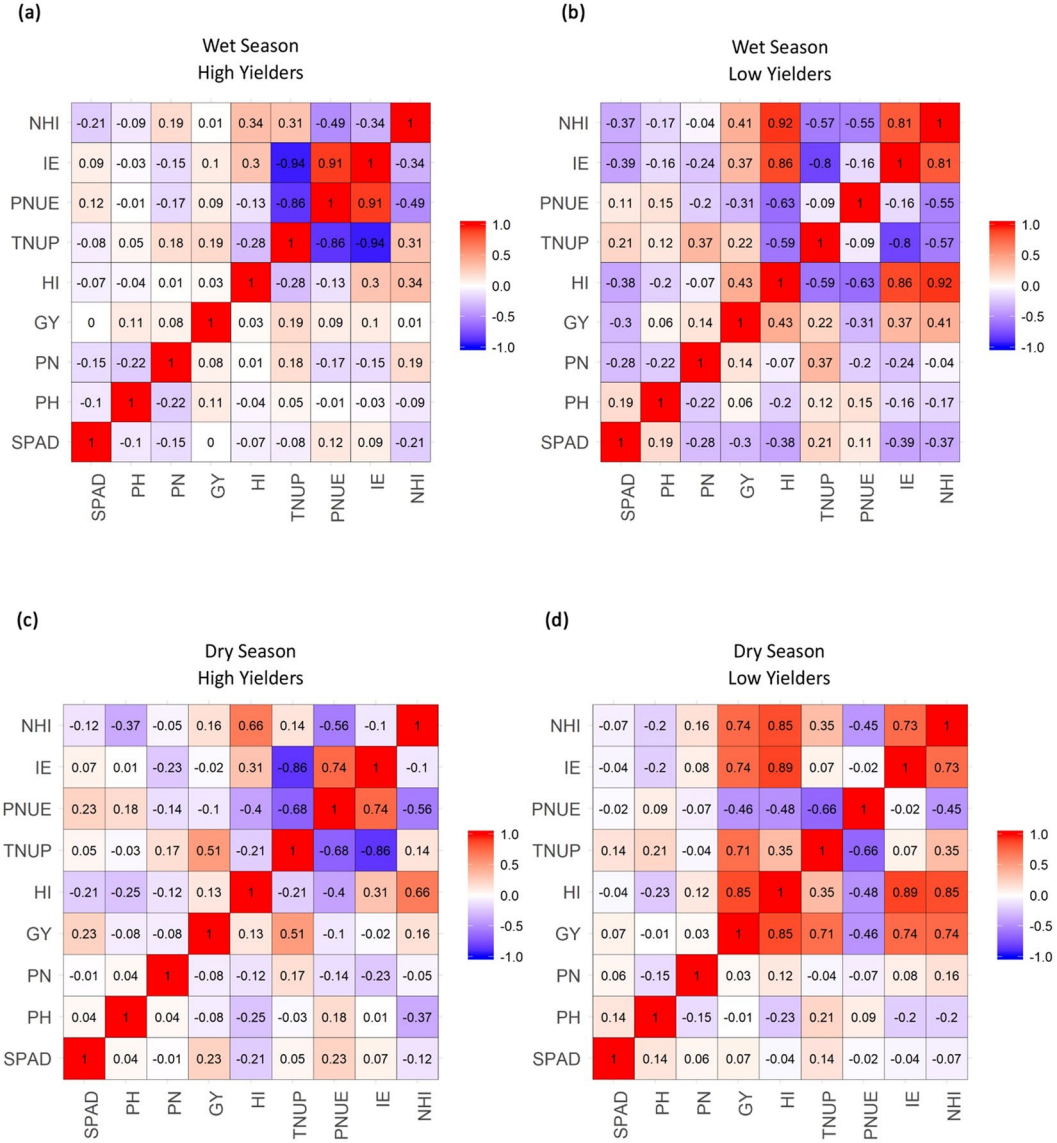
(**a**) Multiple correlations for nine agro-morphological traits/parameters under low N in wet season among high yielders (>3000 kg ha^−1^ - WS); (**b**) Low yielders (<1500 kg (**c**)). Multiple correlations for nine agro-morphological traits/parameters under low N in dry season among high yielders (>2500 kg ha^−1^ - DS) and (**d**) Low yielders (<1500 kg ha^−1^).

To understand the relationship among the traits/parameters and to explore the possibility of using the phenotype data as selection criteria for yield under low N, multiple correlations were performed across genotypes with high and low yields under low N resulting only in obvious significant positive correlations like for grain yield with total N and total dry matter across the seasons. The interesting observation was the wide variation showed for correlation analyses of high and low yielders especially for SPAD and panicles m^−2^ across the seasons (Fig. 2a–d).

### Grouping of germplasm by population structure

A total of 177 alleles were obtained using 50 SSR markers among the 472 genotypes with the average alleles of 3.54 per SSR locus ranging from 2 to 6. The average gene diversity ranged from 0.54 ranging from 0.34 to 0.72 with average PIC value of 0.45 ranging from 0.29 to 0.67 (Supplementary Table S3). The model based simulation of population structure showed the highest value of ΔK = 4 for 472 rice genotypes (Supplementary Fig. S3). The population structure showed four subgroups and the grouping was consistent with clustering based on genetic distance calculated by DARwin 5.0 (Supplementary Fig. S4). AMOVA estimates showed a total variation of 9.66% among four subgroups and 90.34% of variation within subgroups (Supplementary Table S3). The overall F_ST_ value was 0.097 among subgroups and the gene diversity among the subgroups ranged from 0.43 to 0.55 (Supplementary Table S3).

### Moderate level of linkage disequilibrium (LD) and LD decay

At the whole population level, *r* ^2^ values among the 50 SSR pairs ranged from 0.0 to 0.1285 with an average of 0.008. The intrachromosomal *r*^2^ values among all the SSR pairs ranged from 0.0295 to 0.1142. The D′ ranged from 0.02 to 0.19 with an average of 0.085 at the whole population level. SSR pairs with LD based on D′ (P < 0.05) occupied 40% in total population ranging from 32 to 42% across populations. The genetic distance of marker pairs on the same chromosome showed that a mean distance of LD decay in overall population was 56.4 cM with a range of 34.5 to 52.4 cM in across populations.

### MLM identifies potential marker trait associations

The marker-traits associations identified by mixed linear model (MLM) were analyzed over the associations identified by general linear model (GLM). MLM considers both population structure and kinship for association mapping, thus is more suitable for the material of the present study over GLM, where only population structure is considered for analyses. Under low N in wet season, seven marker trait associations with five SSRs under field conditions were identified (RM495, RM22, RM55, RM169 and RM1381). Interestingly, NHI% was associated with four markers (RM495, RM169, RM22 and RM1381). RM22 also had shown association with two morphological traits, plant height and SPAD. HI was found to be associated with RM55. For dry season, grain yield was associated with RM514 and RM507. The total N uptake and IE were found to be associated with RM1381 and RM22, while RM495 was associated with panicles m^−2^. For panicle traits, 21 traits associations with 10 SSRs were observed. RM271 found to be the most promising marker with associations of grain filling (%) of secondary branches, spikelets on primary branches of lower portion, grain filling (%) of lower portion, grains on lower portion and total grains. The length of the panicle was associated with RM507. Spikelets on primary branches of upper portion found to be associated with RM208 and lower portion with RM271. Spikelets on secondary branches of lower portion were associated with RM22 and RM210. The spikelets on branches of lower portion were associated with RM169 and the total spikelets on primary branches with RM154. Grains on primary branches of lower portion were associated with RM22 and on secondary branches of lower portion with RM154. The total grains on branches of lower portion were found to be associated with RM495, on lower portion with RM271 and on primary branches with RM514. The grain filling (%) of secondary branches on upper portion was associated with RM154; on lower portion with RM271 and RM169; of secondary branches with RM271and the total grain filling (%) with RM507. The total grains were associated with RM271, RM455 and RM514. The alleles of RM22 and RM271 have shown positive and negative allelic effects across subgroups (Table 4). The marker trait associations for recommended N were listed in Supplementary Table S4.

**Table 4:** Association of markers with traits/parameters using MLM under field (F) and panicle traits (PT) showing the phenotypic variance (R^2^) and average allele effect (AAE) on the phenotypic traits under low N.

S.No.		Marker	Chr No.	Subgroup	Trait/parameter	P value	R^2^ (%)	AAE	Size (bp)
1	F	RM495	1	1	WS NHI%	3E-02	>20%	16.88	170
2	F	RM495	1	2	DS panicles m^−2^	5E-03	1.37%	32.98	160
3	PT	RM495	1	2	Grains on branches of lower portion	4E-02	2.34%	4.11	170
4	PT	RM154	2	2	Grain filling (%) of secondary branches of upper portion	8E-23	>20%	1.20	190
5	PT	RM154	2	2	Spikelets on primary branches	7E-14	>20%	1.45	183
6	PT	RM154	2	2	Grains on secondary branches of lower portion	1E-02	>20%	1.44	183
7	PT	RM208	2	2	Spikelets on primary branches of upper portion	3E-36	>20%	2.72	173
8	F	RM22	3	2	WS SPAD reproductive stage	4E-03	8.01%	−4.72	205
9	F	RM22	3	2	WS Plant height	2E-03	1.58%	15.80	205
10	F	RM22	3	2	WS NHI%	5E-04	2.15%	−7.23	205
11	F	RM22	3	2	DS IE (kg kg^−1^)	7E-14	11.25%	22.45	194
12	PT	RM22	3	2	Grains on primary branches of lower portion	4E-06	>20%	1.35	205
13	PT	RM22	3	2	Spikelets on secondary branches of lower portion	3E-04	>20%	−28.43	205
14	F	RM55	3	1	WS HI	4E-02	>20%	−2.98	235
15	F	RM514	3	2	DS Grain yield (kg ha^−1^)	9E-21	>20%	1607.25	265
16	PT	RM514	3	2	Grains on primary branches	2E-08	>20%	4.60	259
17	PT	RM514	3	2	Total grains	9E-05	>20%	21.01	259
18	F	RM507	5	2	DS Grain yield (kg ha^−1^)	2E-10	12.89%	586.00	270
19	F	RM507	5	2	DS HI	1E-04	14.14%	3.75	270
20	PT	RM507	5	2	Grain filling (%)	1E-03	2.16%	30.78	265
21	PT	RM507	5	2	Panicle length	9E-03	>20%	7.98	270
22	F	RM169	5	3	WS NHI%	8E-19	>20%	11.09	170
23	PT	RM169	5	2	Spikelets on branches of lower portion	3E-19	>20%	17.39	167
24	PT	RM169	5	2	Grain filling (%) of lower portion	5E-14	>20%	88.95	170
25	PT	RM455	7	2	Total grains	1E-09	>20%	72.30	140
26	F	RM1381	8	3	WS NHI%	5E-10	>20%	49.46	236
27	F	RM1381	8	3	DS Total N uptake	3E-14	>20%	24.41	236
29	PT	RM210	8	2	Spikelet on secondary branches of lower portion	1E-10	>20%	42.90	140
30	PT	RM271	10	2	Grain filling (%) of secondary branches	4E-14	>20%	1.18	115
31	PT	RM271	10	2	Total grains	2E-05	2.25%	−23.52	115
32	PT	RM271	10	2	Spikelets on primary branches of lower portion	3E-04	1.38%	−12.01	115
33	PT	RM271	10	2	Grains on lower portion	3E-03	1.92%	−25.75	115
34	PT	RM271	10	2	Grain filling (%) of lower portion	5E-02	>20%	1.25	101

The identified SSR markers from association mapping of the 427 genotypes were evaluated using existing 218 recombinant inbred lines (RIL) population developed between BPT5204 and Varadhan. BPT5204 is a check variety for yield and quality and Varadhan was found to be promising for yield under low N from our earlier study^19^ and the characters of BPT5204 and Varadhan are given in Supplementary Table S5. Mapping of RIL population showed four SSR markers, RM507 and RM169 (with grain yield), RM495 (with panicles m^−2^) and RM271 (with total grains) found to be associated under low N.

### Novel genomic regions for yield under low N identified through association mapping

Out of 12 associated genomic regions under low N identified in this study, five genomic regions (38.5%) *viz*., RM495, RM154, RM507, RM1381 and RM271 are novel genomic regions for yield under low N. Seven genomic regions (RM208, RM22, RM55, RM514, RM169, RM455 and RM210) were co-localized with reported QTL for NUE and related traits (Supplementary Table S6).

### *Insilico* analysis of identified genomic regions from association mapping

From the analysis of five Mb region spanning each of the associated 12 SSR markers in the rice genome sequence, 103 to 277 candidate genes with an average of 166.5 were noted and a minimum of two candidate genes directly related to N metabolism were observed (Supplementary Table S7).

### Local Linkage map construction of four genomic regions based on map based cloning approach and QTL analyses

**Figure 3:**
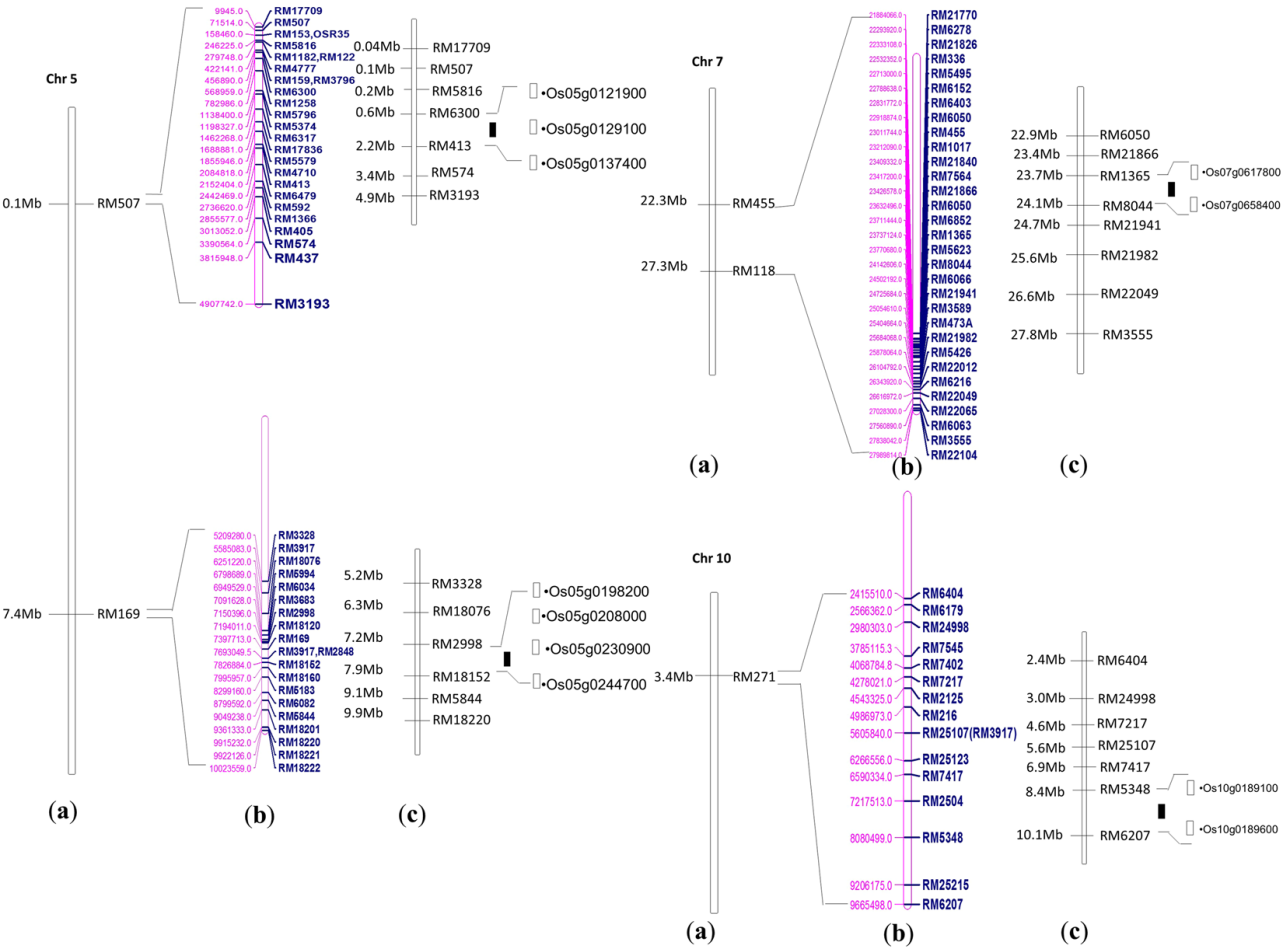
Local linkage maps of four associated genomic regions of chromosomes 5, 7 and 10. Solid bars indicate the position of identified QTL in the present study and bars indicate the candidate genes targeted for expression analyses. (**a**) SSR markers identified through association mapping. (**b**) SSR markers screened for parental polymorphism. (**c**) Local linkage maps with polymorphic SSR markers.

Out of 12 genomic regions identified to be associated with traits of interest under low N, four genomic regions *viz*., 0 to 5 Mb (RM507), 5.1 to 9.5 Mb (RM169) of chromosome 5, 24.7 to 29.5 Mb (RM495-RM118) of chromosome 7 and 1 to 6 Mb (RM271) of chromosome 10 were selected based on the number of the associations in the present study and reported QTL. Following the map based cloning strategy, SSR markers in these four regions were surveyed for their polymorphism between the parents of existing RIL mapping population of BPT5204 (a high yielding variety popular for its quality) and Varadhan (a variety with high yield under low N)^19^. Seven polymorphic SSRs in the region of RM507 out of 23 SSRs, six SSRs spanning RM169 of chromosome 5 out of 19 SSRs, eight SSRs in the region of RM455 of chromosome 7 out of 32 SSRs and seven SSRs spanning RM271 out of 14 SSRs were used for local linkage map construction of BPT5204/Varadhan. QTL analyses for yield under low N showed a major QTL *qGYLN7*, explaining large phenotypic variation (PV) (~20%) located between RM1365 and RM8044 of chromosome 7 (RM455) and another QTL, *qGYLN5-1* (~15% PV) associated with RM18076 and RM2998 of chromosome 5 (RM169). Two minor QTL (~10% PV) *viz*., *qGYLN5-*2 between RM6300 and RM413 of chromosome 5 (RM507) and *qGYLN10* between RM5348 and RM6207 of chromosome 10 (RM271) were also identified (Fig. 3) (Supplementary Table 8).

### Expression analysis of candidate genes in the associated genomic regions with yield under low N

**Figure 4:**
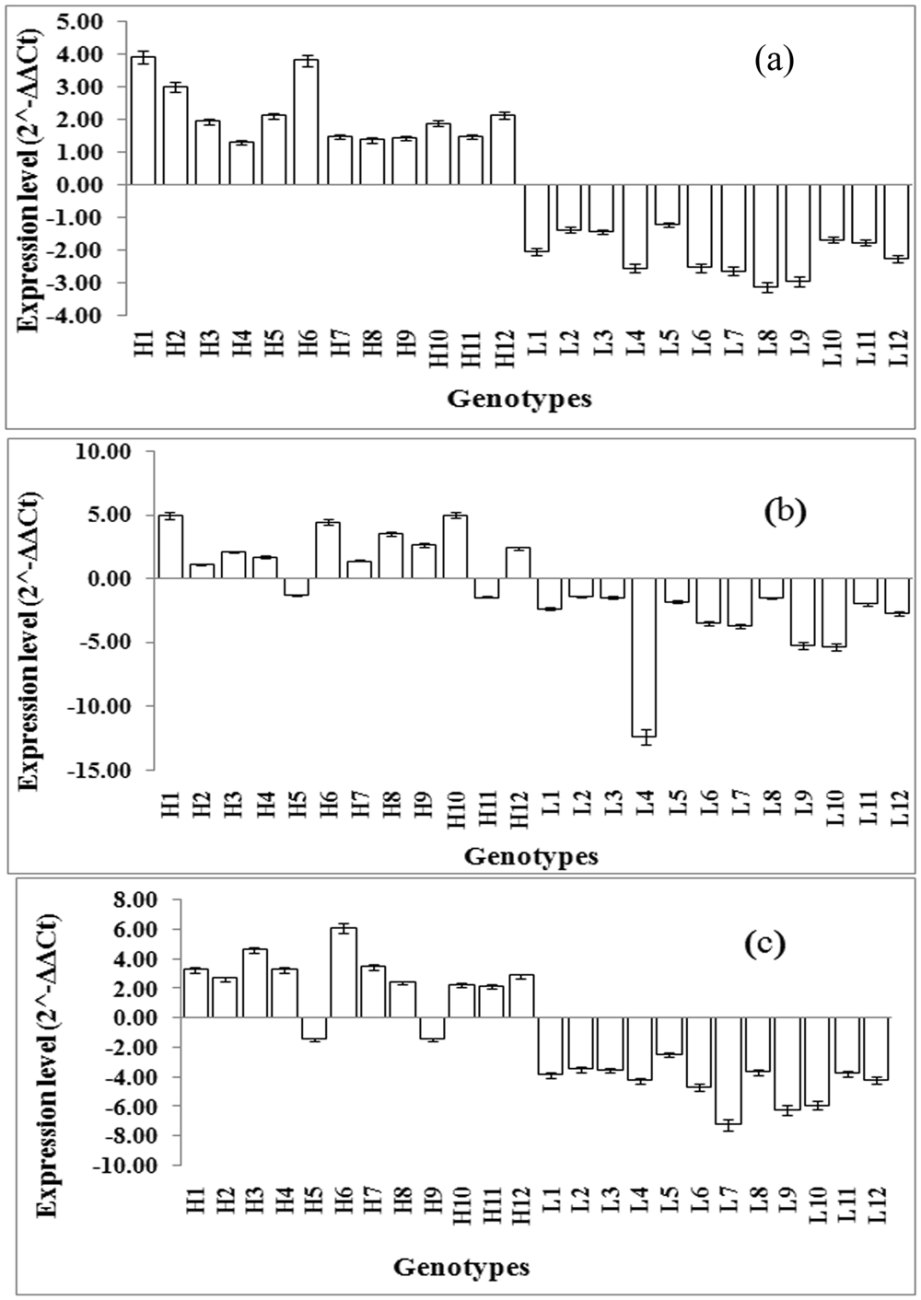
Expression analysis of three candidate genes in 24 genotypes with relative higher yield in 12 genotypes (H1–H12) and 12 genotypes with relative poor yield (L1–L12) under low N; (**a**) alanine aminotransferase; (**b**) 2-oxoglutarate/malate translocator (**c**). Pyridoxal phosphate-dependent transferase.

As per the map based cloning strategy, putative candidate genes were listed using the rice genome sequence in the marker delimited region of identified QTL. For expression analysis, two novel genomic regions and two genomic regions associated with reported QTL were targeted. In *qGYLN7*, 25 putative genes were found in 405.5 kb region. Spanning 899.2 kb region, 34 putative genes were listed in *qGYLN5-1*. In *qGYLN5-2*, 92 putative genes were found in 1.5 Mb region and 52 putative genes were identified in 1.5 Mb region of *qGYLN10* (Supplementary Table S8). Candidate genes associated with N metabolism in the genomic region were only considered for expression analyses *viz*., two in *qGYLN7*, three in *qGYLN5-1*, four in *qGYLN5-2* and two in *qGYLN10* in the present study. After preliminary screening of differential expression of selected 11 candidate genes associated with N metabolism between 12 genotypes with contrasting yield under low N, three genes showed significant differential expression. Further analyses of these three genes in 36 genotypes with higher yield and 12 genotypes with lower yield under low N showed significant increased fold changes of expression for 2-oxoglutarate/malate translocator (Os05g0208000) in *qGYLN5-1*, alanine aminotransferase (Os07g0617800) in *qGYLN7* and pyridoxal phosphate-dependent transferase (Os10g0189600) in *qGYLN10*. The fold changes of 12 genotypes with higher yield under low N was shown in Fig. 4a–c and the fold changes of an additional set of 24 genotypes with higher yield was presented in Supplementary Table S9. For the remaining eight genes, clear differential expression between the genotypes with differential yield was not observed (Supplementary Table S10). Though QTL was identified between RM6300 and RM413, differential expression was not observed for the selected candidate genes between the genotypes with relative high yield and low yield under low N.

### Development of BPT5204 (a mega rice variety) with positive alleles from Varadhan

Using positive marker alleles of RM18076 and RM2998 (2-oxoglutarate/malate translocator), RM1365 and RM8044 (alanine aminotransferase) and RM5348 and RM6207 (pyridoxal phosphate-dependent transferase) associated with grain yield under low N, two lines *viz*., Varadhan/ BPT 5204/6 and Varadhan/BPT 5204/10 were selected from the cross of BPT5204 and Varadhan (Supplementary Table S11). On evaluation in three locations, these two lines have shown relative high yield under 50 kg N ha^−1^ over parents (Supplementary Table S12).

## Discussion

Landraces of rice, the genotypes selected by farmers over time for their grain yield and traits like quality and stress tolerance grown mostly under low or minimum inputs likely to harbour the trait of resource use efficiency, especially the NUE. True to our premise, we could identify >100 genotypes with promising yield under low N in our study with efficient utilization of absorbed N. The wide and variable response to low N in the present study confirms the reported variability of the differential N metabolism capabilities of the individual genotypes. With the utilization of the large number of gentoypes in the present study, we could pin point the traits and mechanism *viz*., grains on secondary branches and partitioning of N into the reproductive parts to be critical for yield under low N.

Variations between wet and dry seasons and their correlations can be attributed to difference of the light, sunshine hours, humidity, rainfall and temperature. The wet season appears to be more vulnerable for low N conditions for plant height, whereas, the tillers, productive tillers and panicle number were relatively reduced more in dry season. While an optimum concentration of N > 35 g/kg reported to activate tillering, concentration less that than could reduce the tillering^20^. Thus the limited translocation of N from culms to leaves leads to the reduction of photosynthates, therefore decrease the translocation of nutrients to developing panicles, thereby reducing the total biomass and yield.

Landraces have general tendency for vigorous vegetative growth, thus several genotypes in the present study were identified with high PNUE indicating their conversion efficiency of the absorbed N into total dry matter, mostly the straw. Genotypes were also identified with high internal use efficiency reflecting the efficiency of assimilated N for grain yield production through the differences in internal N requirements of the individual genotypes for expansion growth, mass accumulation and organ formation^21^. The variability of IE across the genotypes may also be due to the differential rate of radial and axial flow of total N, its distribution to sources and sinks (leaves or roots or panicles) at different stages, remobilization from older organs to active tissues, flag leaf N import/export, leaf senescence patterns, efficiency in converting CO_2_ to carbohydrate and retention of N in the straw at maturity^22,23^.

While expected correlations were obtained with grain yield and total N uptake in the analyses of genotypes as high, moderate and poor yielders, an interesting observation was the high yielders across the season efficiently partition their N into grain yield, whereas the low yielders convert their N for increasing their biomass. In our study, we could identify several genotypes with high NHI% (>70) with higher conversion efficiency. NHI was reported to be around 60% and suggested to be genotype specific in the earlier studies^6,23^. Some land races with higher IE and NHI lodged because of their height and weak culm, thus those landraces were identified only as donors of high IE and NHI. Crosses are being made to get the favourable recombinants using the landraces with undesirable plant type, but positive alleles for NUE.

Distribution of spikelets and grains across the panicle showed the importance of contribution of spikelets and grains on secondary branches of the panicle towards the total grain number under low N. Maximum reduction for the number of spikelets and grains on secondary branches of lower portion under low N suggested the limitation of assimilates for the production of the spikelets and their filling on secondary branches of lower portion under low N. In fact, yield increase in the high yielding varieties of rice under green revolution was credited to the increase of the grain as well as spikelet number on secondary branches which is dependent on the N fertilization^24,25^. Under resource limited conditions, the plant tries to survive and propagate itself by producing the necessary grains, thus grains were mostly observed on primary branches. Even, the grain filling % found to be better under low N owing to the propensity of the panicle for filling of the available spikelets under resource limited conditions. Because of the wide genetic variability of the material of the present study, genotypes with 235 grains per panicle from with maximum number of grains on the secondary branches of lower portion (100) under low N were identified. From the screening of 472 genotypes for the agro-morphological, yield and N related traits and their correlations, we suggest that grains on secondary branches, total N uptake and grain yield to be the selection criterion for promising genotypes under low N.

Association mapping takes advantage of historic linkage disequilibrium *i.e*., the random association of alleles at different loci existing in the germplasm for linking to the phenotypic characteristics, thus in the present study, LD was exploited to identify the genetic associations with yield and related components using SSR markers under low N^13–18^. Many association mapping studies in rice have identified genomic regions associated with germination to HI and also various abiotic and biotic stresses using SSR markers^13,14,26–29^. So far ~192 QTL through biparental mapping populations and eight genomic regions through association mapping have been identified for N related traits under low N^15^. The five novel genomic regions identified for yield under low N despite several reported genomic regions suggests the potential of landraces as new genetic resources for identifying new genomic regions and genes for NUE in rice. The interesting observations for panicle traits were the association of different markers for spikelets and grains on primary and secondary branches of lower and upper branches of the panicle and their filling suggesting the possibility of the pyramiding of the favourable alleles of the markers for yield across the panicle under low N. Another interesting observation is the involvement of a single marker with several panicle traits under low N implying its utility as marker and scope for finding the associated candidate gene linked to the marker.

As followed in map based cloning/positional strategy, the differential expression was observed for 2-oxoglutarate/malate translocator (Os05g0208000), alanine aminotransferase (Os07g0617800) from the co-localized QTL and pyridoxal phosphate-dependent transferase (Os10g0189600) in the novel genomic region. The role of alanine aminotransferase in NUE of rice has already been demonstrated by genetic engineering of alanine aminotransferase with tissue specific expression^30^. The enhanced expression of 2-oxoglutarate/malate translocator gene and its association with yield under low N through the identified QTL is being reported for the first time in the present study. The role of 2-oxoglutarate in GS/GOGAT pathway and cell carbon/nitrogen status is known and 2-oxoglutarate is now being considered as master regulator metabolite^31,32^. Pyridoxal phosphate-dependent enzymes are reported to be primarily involved in the biosynthesis of amino acids and amino acid-derived metabolites^33^. The enhanced expression of the 2-oxoglutarate/malate translocator and pyridoxal phosphate-dependent transferase in genotypes with promising yield under low N need further validation. We have validated only the genes with known function related to nitrogen metabolism from the identified QTL in the present study and the role of all the other genes present in the identified genomic regions is being attempted through further studies. The differential expression of the three candidate genes in the high and poor yielders under low N indirectly confirms the genomic regions identified through association mapping for NUE in rice.

The outcome of present association mapping study was deployed for MAS using associated SSR marker alleles of alanine aminotransferase, 2-oxoglutarate/malate translocator and pyridoxal phosphate-dependent transferase among RIL. Two RILs of Varadhan/ BPT 5204 have shown relative higher yield confirming the efficacy of the identified genomic regions for yield in rice under 50 kg N ha^−1^.

In conclusion, after evaluation of substantial number of genotypes under low N, we have identified >100 rice landraces with relative higher yield under low N as donors in our study and characterized for the higher total N uptake, N translocation into grains and grain yield under low N. Grains on secondary branches, total N uptake and grain yield appears to be the selection criterion under low N. Through association mapping, we could show that use of a minimum set of SSR markers could identify the genomic regions associated with the traits of interest and fine mapped them using map based cloning strategy. As a proof of concept, two novel regions and two regions co-localized with reported QTL were selected, local linkage map constructed with polymorphic SSR markers and QTL for yield under low N were identified in the existing mapping population. Taking the putative candidate genes from the marker de-limited regions, enhanced expression of the three candidate genes was studied in the landraces with promising yield under low N for confirmation. The identified genomic regions were selected using SSR markers in the existing cross made with a popular variety with a check variety carrying the positive alleles and the breeding lines were evaluated across three locations for their performance under low N and two promising lines for yield were identified under low N. The study brings out the possibility of use of landraces as source for NUE by identifying the donors and native genes for yield under low N.

## Methods

### Plant materials

The plant material comprised a set of 472 rice genotypes with landraces and breeding lines and a second set of 218 recombinant inbred lines (RILs) developed between BPT5204 (a check variety for yield and quality) and Varadhan (a check variety for promising yield under low N)^19^ (Supplementary Table S13).

### Phenotyping – Field

The first set of germplasm was screened under low N (without application of N) and recommended N during two consecutive seasons of wet (*Kharif*) 2011 (WS) and dry (*Rabi*) 2012 (DS) at ICAR-Indian Institute of Rice Research (IIRR), Hyderabad, India. The characteristics of the plots and soil during wet and dry seasons (2011 and 2012) were detailed^4^ (Supplementary Table S14). The experiment was conducted in a split plot design, without N application and with N application as main plots and genotypes as subplots in three replications and the fertilizer applications were followed as per earlier studies^4,19^. Nitrogen fertilizer @ 100 kg ha^−1^ was supplied in the form of urea (46.5%) in three equal split applications to the recommended N treatment (at basal, maximum tillering and panicle initiation stages). Phosphorus (@40 kg ha^−1^), potassium (@40 kg ha^−1^) and zinc (@25 kg ha^−1^) were applied to both plots.

One month old seedlings of 472 rice genotypes were transplanted at a spacing of 10 × 20 cm (Supplementary Table S13). From each line, five representative plants were harvested at maturity and were divided into vegetative and reproductive parts, dried and weighed for determining dry matter of various plant parts. Grain and straw yield was adjusted to 14% grain moisture content and expressed in kg ha^−1^. Straw and grain samples were analyzed for N with Kjeldahl method. A total of nine traits/parameters were recorded for morphological, yield and nitrogen content in low and recommended N conditions for wet and dry seasons (Supplementary Table S15). Analysis of the variation for the nine traits/parameters across the genotypes grouped as high yielders (>3000 kg ha^−1^ - WS) (>2500 kg ha^−1^ - DS), moderate yielders (>1500 kg ha^−1^) and low yielders (<1500 kg ha^−1^). For a random subset of 206 genotypes of first set of germplasm, three panicles from plant were collected and characterized for panicle length (from base to the tip) and filled and unfilled spikelets on primary and secondary branches(Supplementary Table S13). The distal half of the panicle was taken as upper half and proximal half was taken as lower portion of the panicle. The grain filling percentage was calculated based on filled spikelets to the total number of spikelets (Supplementary Table S15). The second set of 218 RILs along with the two parents was grown during wet season (*Kharif*) 2015 at low and recommended N plots and data for grain yield was collected.

### Data analysis

NUE indicators have been calculated for low and recommended N individually *viz*., Physiological Nitrogen Use Efficiency (PNUE)^25^, Internal Efficiency^34^ and Nitrogen Harvest Index (%)^23^ (Supplementary Table S15). Two way analysis of variance (ANOVA) and correlations were performed using an open source software R (R Core Team, 2012) with agricolae package. Multiple correlations were performed for the nine traits/parameters across the genotypes grouped as high yielders (>3000 kg ha^−1^ - WS) (>2500 kg ha^−1^ - DS) and low yielders (<1500 kg ha^−1^).

### Association mapping

Total genomic DNA from 472 genotypes and mapping population was isolated using modified protocol^24^. A total of 50 rice microsatellite markers based on simple sequence repeats (SSRs) across the 12 chromosomes were selected for association mapping analysis (Supplementary Table S8). All primer sequences of microsatellites were obtained from http://www.gramene.org/. For amplification of the microsatellite markers, PCR was carried out in Thermal Cycler (Bio-Rad C1000) using 40 ng template DNA, 0.125 mM dNTPs each, 2.5 p moles of forward and reverse primers, 1 U Taq polymerase (Bangalore Genei, India) and 1× buffer (Bangalore Genei, India) in a total volume of 10 µl reaction. The cycling conditions included initial denaturation at 94 °C for 4 min, followed by 30 sec at 94 °C, 30 sec at 55 °C, and 1 min at 72 °C for 35 cycles, followed by a final extension at 72 °C for 7 min. Amplified products were resolved in 8% polyacrylamide gel using mini vertical polyacrylamide gel electrophoresis (PAGE) (CBS Scientific Co. Inc., USA).

### Allelic diversity, population structure and association

The number of alleles, gene diversity (GD), and polymorphism information content (PIC) per locus were calculated with the PowerMarker 3.25^35^. The LD coefficient *r*^*2*^ and D′ of all markers in pairs were evaluated using the software TASSEL 2.1. The LD among SSR markers was determined as per distances indicated in Cornell SSR 2001 map (www.gramene.org). To assess the genetic structure in 472 genotypes, both model and distance based approaches were used. For model based approach, 20 independent simulations were run for each K (from 1 to 10) with burn in length of 100,000 and a model for admixture and correlated allelic frequencies using Structure 2.3.4 program^36^. To determine the K value, the LnP(D) value in the Structure output and Evanno’s Δ between successive K were used^37^. Based on highest ΔK of the data K = 4 clusters of genotypes, Q matrix was calculated. The hierarchical distribution of the molecular variance (AMOVA) within and between subgroupss defined by Structure and pairwise Wright’s fixation index (F_ST_) values were assessed using Arlequin ver.3.11 (cmpg.unibe.ch/software/arlequin3/). For distance based approach, an unweighted neighbor-joining (NJ) tree was constructed based on dissilimilarity matrix using a shared allele index with DARwin 5.0 (http://darwin.cirad.fr/darwin).

Association between SSR markers and phenotypic traits were analyzed by TASSEL 3.0. For each SSR locus, rare alleles in the population (defined as alleles with frequency <5%) were treated as null alleles. Two statistical models (GLM and MLM) were used to evaluate the effects of population structure (Q) and relative kinship (K) for marker-trait associations. The P value (0.05) was used to identify statistically significant loci. A false discovery rate (FDR) of 0.05 was used as a threshold for significant associations as per Benjamini and Hochberg (1995) correction method^38^. The allelic effect of each locus (average allelic effect AAE) associated with a given trait was estimated by comparing the mean phenotypic data for that trait with respect to each allele to the phenotypic data of the null allele (a_i_ = phenotypic effect of the allele i = ∑x_ij_/n_i _− ∑N_k_/n_k_; x_ij_ = phenotypic measurement values of j variety carrying the allele i; n_i_ = the number of materials carrying the allele of i, N_k_ = phenotypic value of the variety of k carrying null allele, n_k_ = the number of materials for the null allele^38^. The four subgroups were analyzed for the marker trait association analysis with phenotypic variance >1% and AAE > 1% (positive or negative) to identify significant associated genomic regions.

### *Insilico* analysis of identified genomic regions from association mapping

Five Mb region spanning each of the 12 associated SSR markers was analyzed for the reported putative candidate genes (http://www.ncbi.nlm.nih.gov/nuccore/JN193288.1) (Supplementary Table S7).

### Mapping

Out of 12 significant associated genomic regions, four genomic regions were selected for validation based on their association with the traits in the present study and reported QTL (www.gramene.org). Based on the position of associated genomic regions around ~5 Mb was targeted for selection of SSR markers. A total of 88 SSR marks comprising 23 SSRs for RM507 and 19 SSRs of RM169 of chromosome 5, 32 SSRs for RM455-RM118 of chromosome 7 and 14 SSRs for RM271 of chromosome 10 were surveyed for polymorphism between the parents *viz*., BPT5204 and Varadhan (Supplementary Table S8). The polymorphic markers were screened in RIL population for local linkage map construction. From the genotypic and phenotypic data of RIL population, four local linkage maps constructed using Joinmap 4.1 and QTL for grain yield were identified using MapQTL 6^40^.

### Validation through expression analysis

Out of 22 putative genes found in 405.5 kb region in *qGYLN7*, 34 putative genes spanning 899.2 kb region in *qGYLN5-1*, 92 putative genes found in 1.5 Mb region in *qGYLN5-2*, and 52 putative genes identified in 1.5 Mb region of *qGYLN10*, 11 putative candidate genes associated with nitrogen metabolism were targeted for expression analyses. In *qGYLN5-2* (RM507), three candidate genes *viz*., Os05g0121900 - Phosphate/phosphoenolpyruvate translocator protein-like; Os05g0129100 Aminotransferase class-III family protein; Os05g0137400 - Aspartic protease precursor; in *qGYLN5-1* (RM169), four candidate genes *viz*., Os05g0198200 Glutaredoxin-like; Os05g0208000 - 2-oxoglutarate/malate translocator; Os05g0230900 - Glyoxalase I; Os05g0244700 Aminotransferase, class IV family protein and in *qGYLN7* (RM495-RM118), two candidate genes Os07g0658400- ferredoxin-dependent glutamate synthase; Os070617800 Alanine aminotransferase; in *qGYLN10* (RM271), two candidate genes Os10g0189100 - Phosphoglucomutase, chloroplast precursor (EC 5.4.2.2); Os10g0189600, Pyridoxal phosphate-dependent transferase were selected (http://www.ncbi.nlm.nih.gov/nuccore/JN193288.1). Based on the sequences of putative candidate genes along with their upstream regions (1 kb), two to six primer pairs were designed per gene ensuring the coverage of complete gene along with upstream region using Primer 3.0 software (http://frodo.wi.mit.edu) and synthesized at Integrated DNA Technologies Inc., (Iowa City, IA) (Supplementary Table S8).

Panicle samples of first set of 12 genotypes along with two parents of the mapping population and a second set of 48 genotypes during booting stage were collected and immediately frozen in liquid nitrogen for RNA isolation. Total RNA was isolated using Trizol reagent (Invitrogen, USA) and the quality of the RNA was assessed using Nanodrop^®^ ND1000 spectrophotometer (Thermo Scientific, USA). RNA samples were treated with RNAse free DNAse (Invitrogen, USA). Approximately 1 µg of total RNA from each sample was used as template for the first-strand cDNA synthesis, using Superscript III reverse transcriptase (Invitrogen, USA). qRT-PCR was performed using Applied Biosystems 7500 Real Time PCR (Life Technologies, USA), in a final volume of 20 μl, containing 10 μl of Platinum^®^ SYBR^®^ Green qPCR Super Mix (Invitrogen, USA) with 500 nM each of forward and reverse primers and 20 ng of the cDNA samples. The real time PCR cycling conditions included a pre-incubation at 50 °C for 2 min and denaturation at 95 °C for 10 min followed by 40 cycles of denaturation at 95 °C for 15 s and annealing and extension at 60 °C for 1 min. qRT-PCR was performed as three biological replicates. Samples were run in duplicates on the same plate along with controls set up for each sample in duplicate using 18 s RNA gene for normalization of gene expression. The data were analyzed using the 7500 Sequence Detection Software (Applied Biosystems, USA) with default baseline and threshold. The relative expression levels of genes were calculated using the 2^−∆CT∆CT^ method, which represents the difference of CT between the control products and the target gene products. Recommended N situation was taken as control and low N was considered as treated. Initially all the primers were checked for the differentially expression in 12 genotypes and the three genes differentially expressed in the preliminary analyses were screened in 48 genotypes.

### Development of BPT5204 with positive alleles from Varadhan

Based on the markers associated with three candidate genes *viz*., RM18076 and RM2998 for 2-oxoglutarate/malate translocator, RM1365 and RM8044 for alanine aminotransferase and RM5348 and RM6207 for pyridoxal phosphate-dependent transferase, selections were made in the segregating population of BPT5204/Varadhan applying MAS and two stable lines (F_6_-_7_) were identified. Two of the promising lines (BPT5204/Varadhan/6 and BPT5204/Varadhan/10) were evaluated under trial entitled “Evaluation of Radiation and Nitrogen use efficient promising rice genotypes” under All Indian Coordinated Rice Improvement Program(AICRIP) at three locations across India with two N treatments (50 kg ha^−1^ and100 kg ha^−1^) for two years^40,41^.

## Electronic supplementary material

Supplementary Table S1

Supplementary Table S2

Supplementary Table S3

Supplementary Table S4

Supplementary Table S5

Supplementary Table S6

Supplementary Table S7

Supplementary Table S8

Supplementary Table S9

Supplementary Table S10

Supplementary Table S11

Supplementary Table S12

Supplementary Table S13

Supplementary Table S14

Supplementary Table S15

Supplementary figures 4
