# Supplementary material for: Identification of rice landraces with promising yield and the associated genomic regions under low nitrogen
Source: Sci Rep. 2018 Jun 15;8:9200. doi: 10.1038/s41598-018-27484-0 (PMC6003918; doi:10.1038/s41598-018-27484-0)

**Identification of rice landraces with promising yield and the associated genomic regions under low nitrogen**

I Subhakara Rao, C N Neeraja*, B Srikanth, D Subrahmanyam, K N Swamy, K Rajesh, P

Vijayalakshmi,T Vishnu Kiran, N Sailaja, P Revathi, P Raghuveer Rao, L V Subba Rao, K Surekha, V Ravindra Babu, S R Voleti

**Supplementary Figure S1.** Frequency of the 472 genotypes for 9 agro-morphological traits/parameters under low and recommended N across wet and dry seasons. The vertical line indicates the grand mean across low and recommended N. (a) SPAD: soil-plant analyses development at RS: Reproductive stage;(b) Plant height; (c) PN; Panicles m-2; (d) Grain yield kg ha-1; (e) Harvest index; (f) Total nitrogen uptake kg ha-1;

(g) PNUE: Physiological N use efficiency kg kg-1; (h) IE: Internal efficiency kg kg-1; (i) NHI: N harvest index

**(a)**


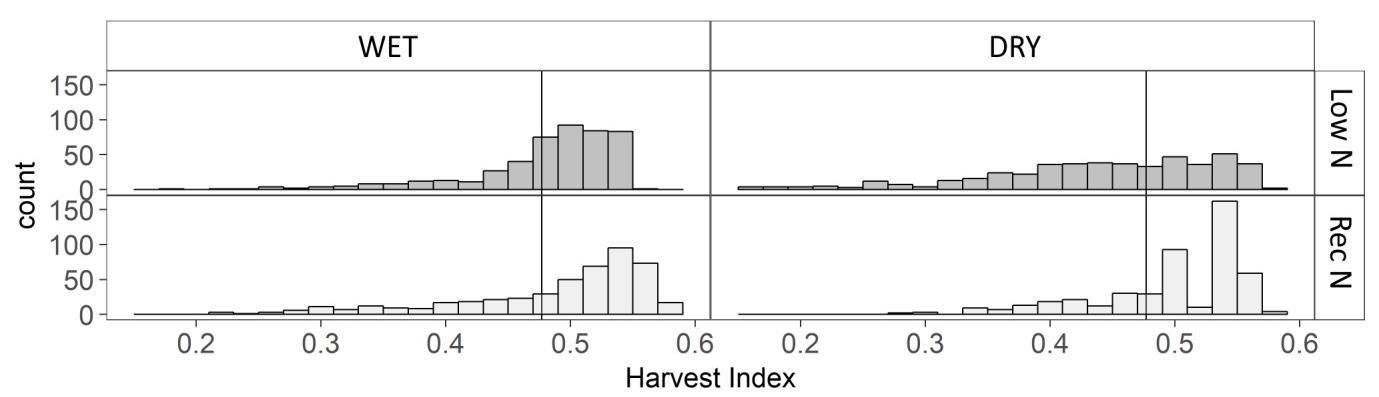

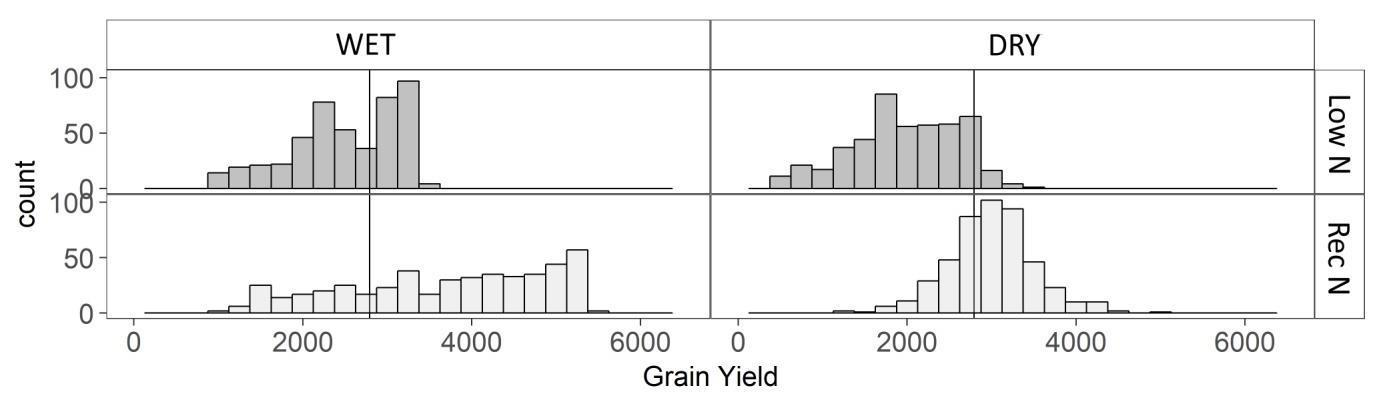

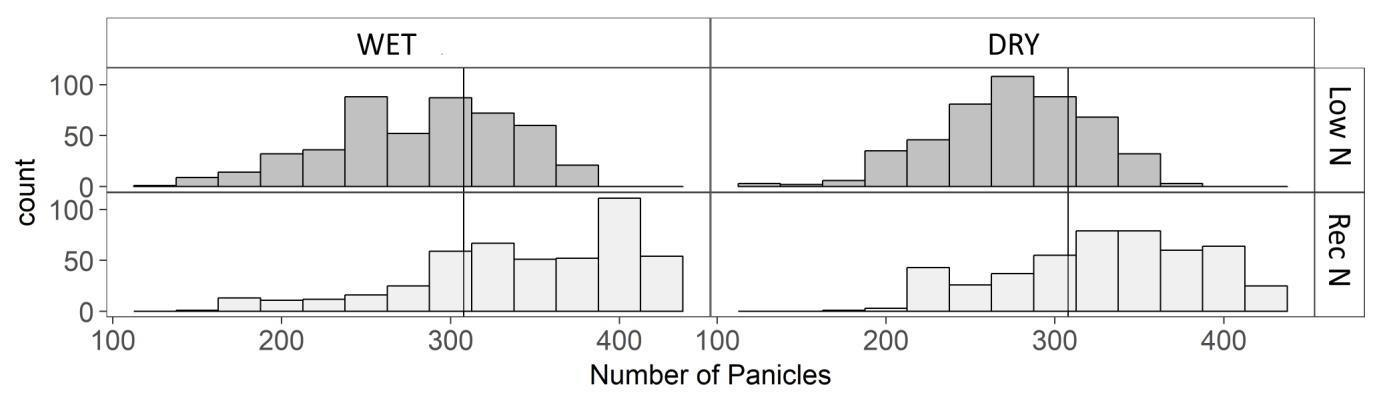

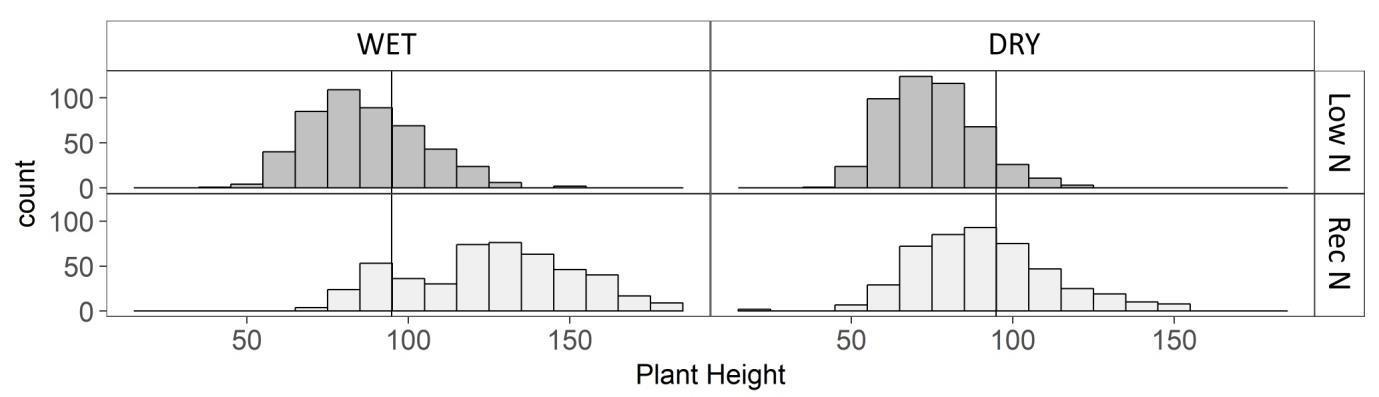

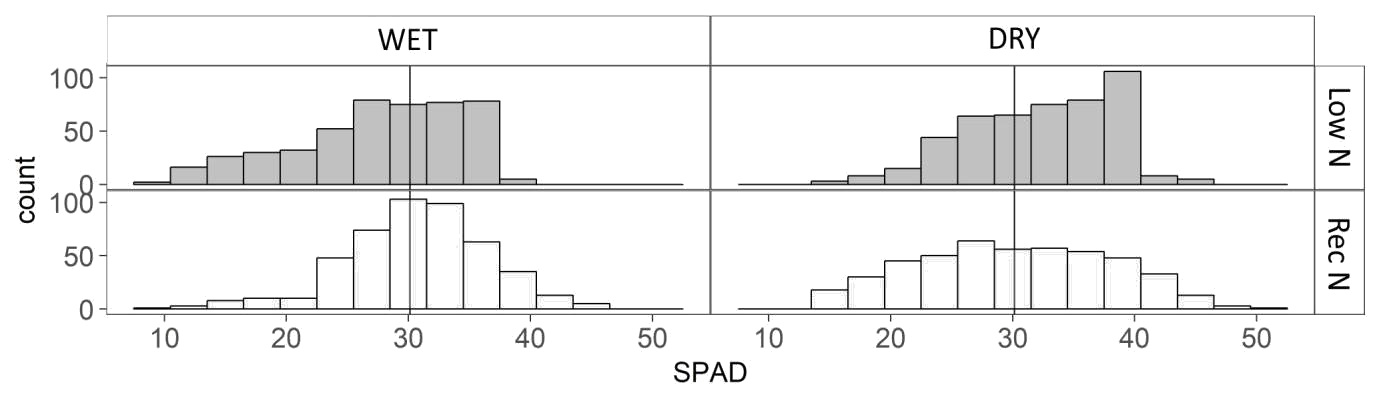


**(b)**

**(c)**

**(d)**

**(e)**

**(f)**

Total Nitrogen Uptake

**(g)**

**(h)**


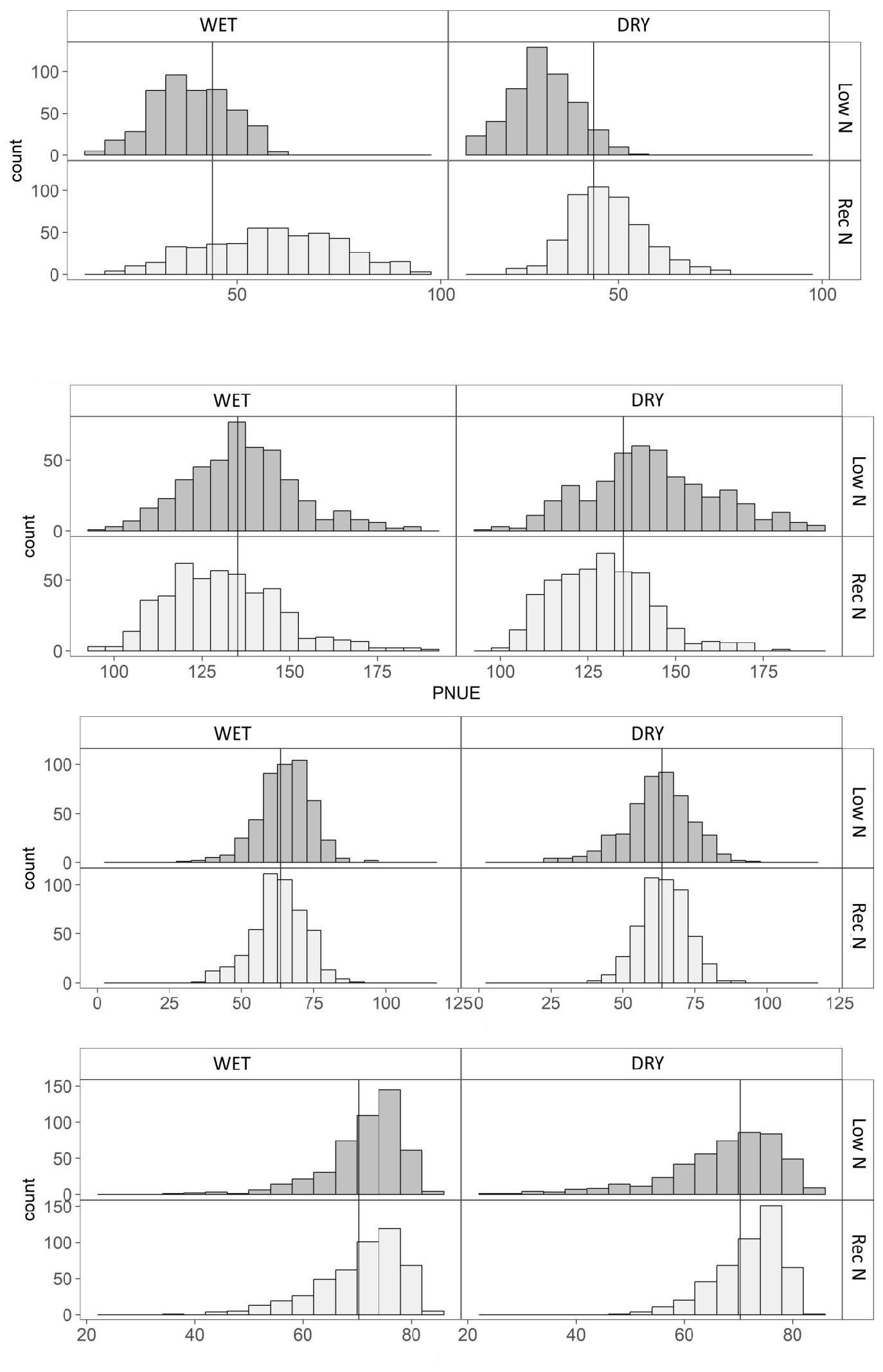


**(i)**

IE

NHI

**Supplementary Figure S2. a.** Representation of variation of SPAD-Vegetative stage; NT:

number of tillers per hill, NPT: number of productive tillers per hill, SW: straw weight kg ha-

1, TDM: total dry matter kg ha-1, NG: N in grain kg ha-1 and NS: N in straw kg ha-1in high yielders (>2500 kg ha-1 - DS), moderate yielders (>1500 kg ha-1) and low yielders (<1500

kg ha-1) in dry season **b.** in wet season **c.** Representation of variation of straw yield (SY) kg ha-1 in wet and dry seasons and **d.** Representation of variation of total dry matter (TDM) kg ha-1 in wet and dry seasons. Each bar represents the mean of 3 independent replications ± STDEV


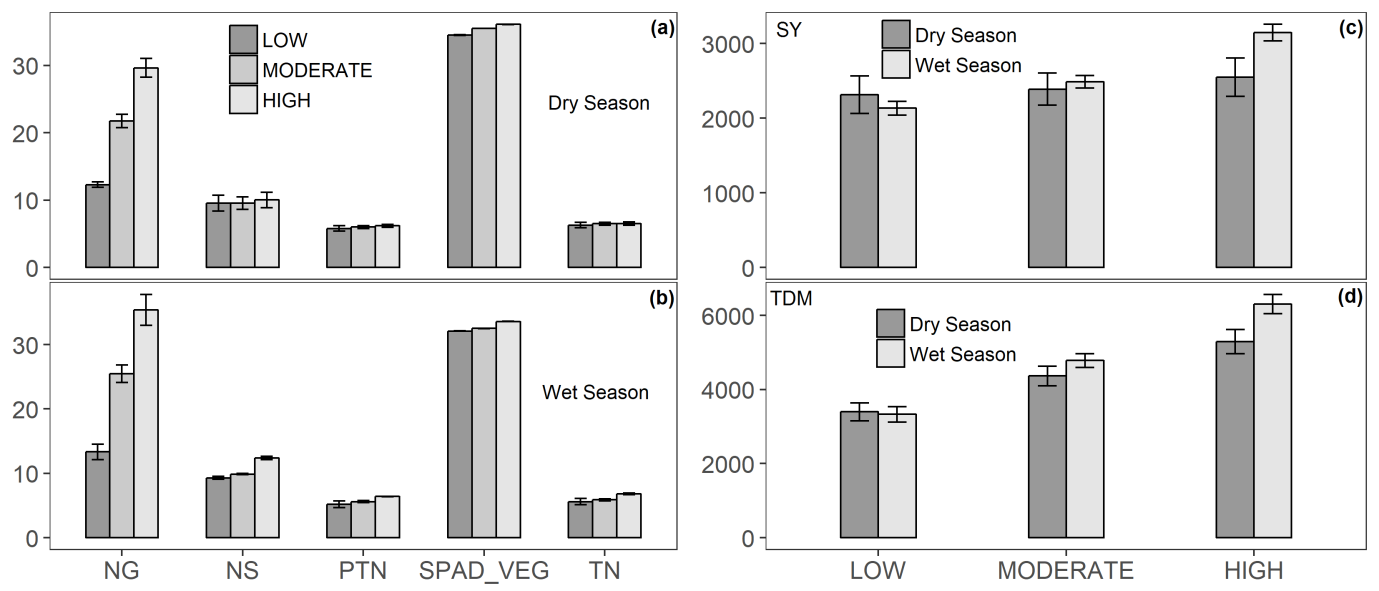


**Supplementary Figure S3.** Population structure analysis of 427 genotypes using model based simulation of population structure using Structure 2.3.4


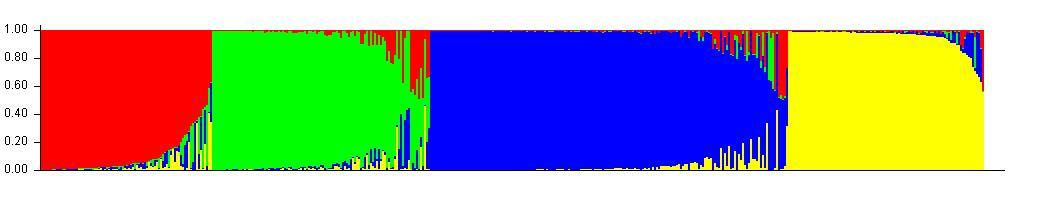


**Supplementary Figure S4.** UPGMA tree of 472 genotypes based on Nei’s genetic distance

(DARwin 5.0)


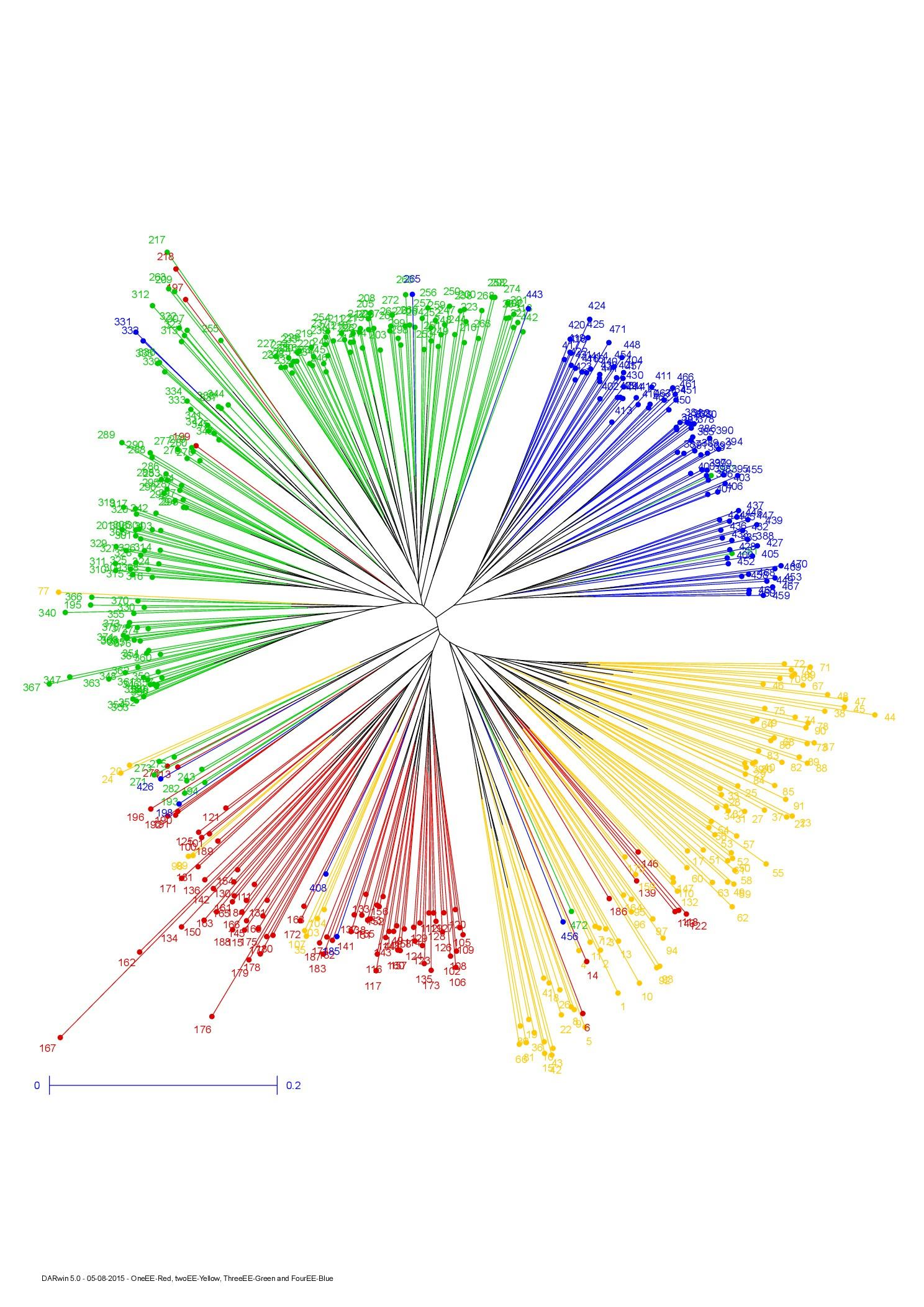

Supplement: Supplementary file 16 — Supplementary figures 4 [file 41598_2018_27484_MOESM16_ESM.docx]
